# A Prognostic Autophagy-Related Long Non-coding RNA (ARlncRNA) Signature in Acute Myeloid Leukemia (AML)

**DOI:** 10.3389/fgene.2021.681867

**Published:** 2021-06-30

**Authors:** Chunxia Zhao, Yulu Wang, Famei Tu, Shuai Zhao, Xiaoying Ye, Jing Liu, Juan Zhang, Zifeng Wang

**Affiliations:** ^1^School of Nursing, Nanchang University, Nanchang, China; ^2^Department of Integrated Oncology, Center for Integrated Oncology, University Hospital Bonn, Bonn, Germany; ^3^Department of Nursing, The First Affiliated Hospital of Nanchang University, Nanchang, China; ^4^Department of Anesthesiology, University Hospital Bonn, Bonn, Germany; ^5^Department of Hematology, Shangrao People’s Hospital, Shangrao, China

**Keywords:** lncRNAs, autophagy, cancer genome atlas, Kyoto encyclopedia of genes and genomes, prognosis, signature, acute myeloid leukemia

## Abstract

**Background:**

Some studies have proven that autophagy and lncRNA play important roles in AML. Several autophagy related lncRNA signatures have been shown to affect the survival of patients in some other cancers. However, the role of autophagy related lncRNA in AML has not been explored yet. Hence, this study aims to find an autophagy related lncRNA signature that can affect survival for AML patients.

**Method:**

A Pearson correlation analysis, a Kaplan–Meier survival curve, a univariate cox regression, and a multivariate cox regression were performed to establish an autophagy related lncRNA signature. A univariate cox regression, a multivariate cox regression, a Kaplan–Meier survival curve, and a ROC curve were applied to confirm if the signature is an independent prognosis for AML patients. The relationship between the signature and the clinical features was explored by using a *T* test. Gene Set Enrichment Analysis (GSEA) was used to investigate the potential tumor related pathways.

**Results:**

A four-autophagy related lncRNA (MIR133A1HG, AL359715.1, MIRLET7BHG, and AL356752.1) signature was established. The high risk score based on signature was related to the short survival time of AML patients. The signature was an independent factor for the prognosis for AML patients (HR = 1.684, 95% CI = 1.324–2.142, *P* < 0.001). The signature was correlated with age, leukocyte numbers, and FAB (M3 or non-M3). The P53, IL6/JAK/STAT3, TNF-α, INF-γ, and IL2/STAT5 pathways might contribute to the differences between the risk groups based on signature in AML.

**Conclusion:**

The four autophagy related lncRNAs and their signature might be novel biomarkers for predicting the survival of AML patients. Some biological pathways might be the potential mechanisms of the signature for the survival of AML patients.

## Introduction

Acute myeloid leukemia (AML) accounts for 65.7% of all acute leukemia (AL) cases and has a higher incidence in male patients (Dores et al., 2012). The incidence rate of AML is 4.3 per 100,000 cases annually and a survival rate of 5 years is achieved by 24% of the patients in the United States (US) (Shallis et al., 2019). This high incidence is related to increasing age and the median age at diagnosis is 68 years (Shallis et al., 2019). Many gene mutations including signaling and kinase pathways (FLT3, KRAS, NRAS, KIT, PTPN11, and NF1), epigenetic modifiers (DNA methylation and chromatin modification), nucleophosmin (NPM1), transcription factors (CEBPA, RUNX1, and GATA2), tumor suppressors (TP53), spliceosome complex (SRSF2, U2AF1, SF3B1, and ZRSR2), and cohesin complex (RAD21, STAG1, STAG2, SMC1A, and SMC3) play important roles in pathogenesis, prognosis, and therapy (DiNardo and Cortes, 2016). However, the etiologies of AML have not been completely understood yet. Improving the treatment of AML is still a great challenge due to the complexity and heterogeneity of AML patients, especially of relapsed and refractory patients. Nevertheless, many advanced and targeted agents emerged recently. Thus, our study focused on finding potential new biomarkers for prognosis and possible therapeutic strategies.

Autophagy is a cellular self-digestion process in which long-lived proteins and damaged organelles are transferred to lysosomes and finally are degraded by lysosomal hydrolases (Li et al., 2020). Autophagy is divided into three forms, including macroautophagy, microautophagy, and chaperone-mediated autophagy (Mizushima and Komatsu, 2011). Non-canonical autophagy pathways play an essential role in AML differentiation (Jin et al., 2018). Two autophagy related genes, ATG7 and LC3, were found to have lower expression in AML patients than in the subjects in the control group (Mohamadimaram et al., 2019). Knockout ATG5 and ATG7 were able to block autophagy, lengthening the survival time of leukemic mice and impairing the ability of leukemia-initiating cells (Sumitomo et al., 2016). Loss of ATG7 improved the outcome of chemotherapy and prolonged the survival of AML mice when compared to control mice (Piya et al., 2017). Hence, autophagy is important for the growth of AML and can change the AML sensitivity of chemotherapy.

Long non-coding RNAs (LncRNA) are types of RNA whose length exceeds 200 nucleotides. LncRNAs are not translated into proteins and account for the largest percentage of non-coding RNAs (ncRNA) (Charles and Eichhorn, 2018). LncRNA ANRIL promote the proliferation of AML cells via the cell glucose metabolism of AdipoR1/AMPK/SIRT1 (Sun et al., 2018). LncRNA NR-104098 hampers the growth of AML cells and promotes the differentiation of AML cells *in vitro* (Feng et al., 2020). LncRNA HOTTIP promotes the proliferation and cell cycle of AML cells by regulating the expression of the DDA1 gene by sponging microRNA-608 (Zhuang et al., 2019). Many lncRNAs have been proven to regulate the growth of AML cells via different pathways. However, to date, no more than 3% of the lncRNA functions have been discovered (Robinson et al., 2020). Thus, further studies are needed to find the lncRNAs that might regulate the growth of AML cells.

## Materials and Methods

### TCGA Data of AML Patients

The TCGA gene expression data (workflow type: HTSeq – FPKM) of the AML patients and the related TCGA clinical data were acquired from UCSC Xena^1^. We selected the cohort named GDC TCGA Acute Myeloid Leukemia (LAML), which contained 151 samples from 151 patients as research subjects. The name of the autophagy gene was taken from the human autophagy database (HADb)^2^.

### Autophagy Related lncRNA

Autophagy related lncRNAs were identified by using a Pearson correlation analysis between lncRNAs and autophagy-related genes. The results were considered significant when they meet the criterion (the correlation coefficients | R| > 0.6 and *p*-values < 0.05).

### Construction of the Autophagy Related lncRNA Signature

A Kaplan–Meier survival curve and a univariate cox regression were performed with a statistical significance *p* value of less than 0.01. According to the criterion mentioned above, most autophagy-related lncRNAs were excluded and the 21 remaining autophagy-related lncRNAs were related to the survival of AML patients.

The best autophagy related lncRNA prognostic signature was selected according to the lowest Akaike information criterion (AIC) value. The risk scores of the signature were calculated by using the following formula: (coefgene 1 × expgene 1) + (coefgene 2 × expgene 2) + … + (coefgene n × expgene n). Herein, expgene represents the expression of lncRNA. Coefgene represents the value of the correlation coefficients in the multivariate cox regression analysis for the lncRNAs. Then, an autophagy-related lncRNA signature was finally established. The cutoff value of the high risk group and the low risk group was the median value of the risk score. A Cox regression, a Kaplan–Meier survival curve, and a ROC curve for the signature were performed to confirm that the signature is an independent factor for the prediction of the survival time of AML patients.

### Gene Set Enrichment Analysis

Gene Set Enrichment Analysis (GSEA) is a computational method that determines whether *a priori* defined set of genes shows statistically significant, concordant differences between two biological states (e.g., phenotypes). Patients were divided into a high and a low risk group based on the risk score in the signature and the cutoff is the median value. Then GSEA was performed to explore the pathways according to their differentially expressed genes in the different groups. And the pathways were selected based on the condition [*p* < 0.05 and false discovery rate (FDR) < 0.25].

## Results

### A Four-Autophagy Gene Related lncRNAs Signature in AML Patients

**FIGURE 1:**
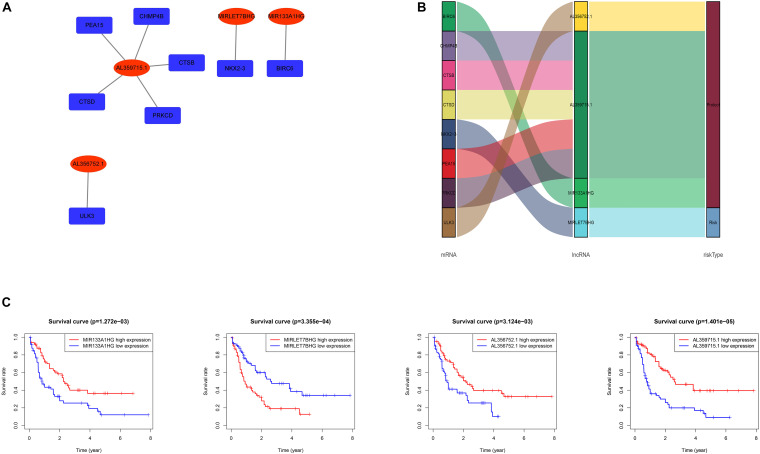
**(A)** Network of the four lncRNAs with co-expressed autophagy-related genes in AML. The red nodes represent the lncRNAs and the deep blue nodes represent the autophagy-related genes. **(B)** The Sankey diagram shows the correlation between autophagy-related genes, autophagy-related lncRNAs and the risk type. The left box represents the autophagy-related genes, the middle box represents the lncRNA, and the right box represents the risk type (favorable/unfavorable). **(C)** Kaplan–Meier survival curves for the four lncRNAs in AML.

The data of the lncRNA and autophagy gene expression of AML patients was extracted from the TCGA database. A total of 151 samples from 151 patients was included in the current study. Firstly, the Pearson correlation method was applied and 866 lncRNAs that were related to the autophagy gene were found based on | R| > 0.6 and *p*-value < 0.05. Then Kaplan–Meier survival curves and univariate Cox regression analysis were performed at the same time to assess gene expression and clinical survival data. 21 lncRNAs were found to be statistically significant as shown in Table 1. A Multivariate cox regression analysis was performed afterward to select the best lncRNA signature. According to the lowest Akaike information criterion (AIC) value, a four-autophagy gene related lncRNA signature was chosen (see Table 2). In Figure 1A, Cytoscape 3.8.2 software was used to visualize the co-expression of the four lncRNAs and autophagy-related genes of AML patients. The Sankey diagram in Figure 1B shows the correlation between the autophagy gene and the lncRNAs and the relationship between the lncRNAs and the risk types. The Impact of the lncRNAs on the patient’s survival was analyzed with the Kaplan–Meier method (see Figure 1C). Figures 1B,C show that MIR133A1HG, AL359715.1, and AL356752.1 are favorable factors associated with a longer survival time for high expression patients than for low expression patients. On the other hand, the left lncRNA (MIRLET7BHG) is associated with a poorer probability of survival for high expression patients compared to the probability for low expression patients, indicating that it is a poor risk factor. According to these results, the four lncRNAs were used to form a signature which might be a risk factor predicting the survival of AML patients from the TCGA database.

**TABLE 1:** Results of 21 lncRNAs from Kaplan–Meier survival curve and Univariate Cox regression in AML patients.

Autophagy gene related lncRNA	Kaplan–Meier survival curve	Univariate Cox regression		
		
	*P* value	HR	*P* value	Adjusted *P* value
AC004492.1	0.000	0.544	0.001	0.016
LINC00899	0.002	1.405	0.000	0.000
AC005070.3	0.007	0.464	0.001	0.016
AC002553.2	0.005	0.510	0.003	0.027
AC007000.3	0.005	0.605	0.007	0.030
HOXA10-AS	0.009	1.329	0.001	0.016
AC127496.5	0.006	0.634	0.009	0.030
MIR133A1HG	0.001	0.392	0.000	0.000
AL121672.3	0.001	1.464	0.009	0.030
ITGB2-AS1	0.000	1.318	0.001	0.016
AL591848.4	0.000	0.654	0.000	0.000
NADK2-AS1	0.005	0.470	0.000	0.000
AL356752.1	0.003	0.619	0.005	0.030
MIRLET7BHG	0.000	1.364	0.003	0.027
AC110792.3	0.000	0.509	0.001	0.016
LINC02175	0.002	0.651	0.001	0.016
AC078860.1	0.005	0.637	0.003	0.027
AC027018.1	0.001	0.574	0.001	0.016
AC022726.1	0.010	0.691	0.005	0.030
AL359715.1	0.000	0.465	0.000	0.000
LINC01503	0.003	1.462	0.007	0.030

**TABLE 2:** Information for four lncRNAs from multivariate Cox regression.

LncRNA	coef	HR	HR.95L	HR.95H	*P* value	Adjusted *P* value
MIR133A1HG	–0.556	0.573	0.356	0.922	0.022	0.088
AL359715.1	–0.440	0.644	0.395	1.050	0.077	0.088
MIRLET7BHG	0.264	1.302	1.030	1.644	0.027	0.088
AL356752.1	–0.381	0.683	0.472	0.990	0.044	0.088

### The Prognosis Function of the Autophagy-Related lncRNA Signature for the AML Patients’ Survival

**FIGURE 2:**
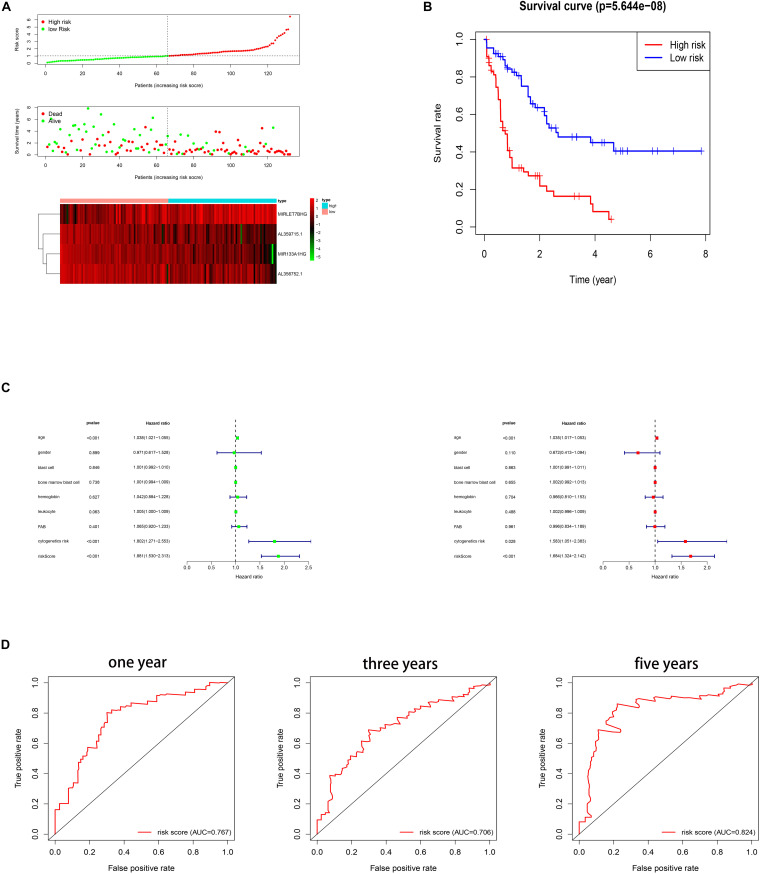
**(A)** Risk scores and survival status of AML patients. Heat map depicting the different expressions of the four lncRNAs in AML patients. **(B)** Kaplan–Meier survival curve for the signature. **(C)** Cox regression. **(D)** ROC curves for 1 year, 3 years, and 5 years.

Several methods were performed to assess the survival predicting ability of the four autophagy related gene lncRNAs signature. Firstly, the patients were divided into a low and a high risk group based on their risk scores of the signature, which were calculated by using the formula mentioned in the methods part. The cut off is the median value of the risk score. As shown in Figure 2A, high risk score patients show a higher death rate and a shorter survival time than the low risk score patients. The heat map indicates the different expression of the four lncRNAs in AML patients, decreasing lncRNAs (MIR133A1HG, AL359715.1, and AL356752.1) and increasing lncRNA (MIRLET7BHG) gene expression in the high risk group. Furthermore, a Kaplan–Meier survival curve was used to investigate the signature in relation to the OS time. The high risk group had a shorter OS time than the low risk group (see Figure 2B). Then, in Figure 2C, univariable and multivariable cox regressions were applied. In univariable cox regression analysis, *p* values less than 0.05 were found in some clinical features, including age, cytogenetic risk, and risk score. Therefore, age, cytogenetic risk, and risk score are prognosis factors. Meanwhile, the same results were predicted in the multivariable cox regression. Thus, age, cytogenetic risk, and risk score are independent prognosis factors. In addition, the ROC curve shows that AUC values were 0.767, 0.706, and 0.824 for 1 year, 3 years, and 5 years, respectively (Figure 2D). Herein, this signature was a reliable independent prognosis factor for AML patients. In conclusion, the signature might predict the survival time of AML patients.

### Correlation Between the Signature and the Clinical Features

To explore the relationship between the signature and the clinical features, a *T* test was applied to compare the risk score of signature in each group. We divided each clinical feature (blast cell, bone marrow blast cell, hemoglobin, and leukocyte) into a high and a low group based on their median value. We set patients whose value is less than their cut off values as the low group and the remaining patients were assigned to the high group. The FAB was grouped into the M3 group and the non-M3 group. Due to the absence of some clinical information, only 127 patients were included here. As shown in Table 3, age, leukocyte, and FAB group had a significant difference (*p* value < 0.05). High age and high leukocyte numbers were related to the high risk score of the signature. The M3 group showed a lower risk score than the non-M3 group.

**TABLE 3:** The relationship between the signature and clinical features.

Clinical feature	Group	*n*	Mean	SD	*t*	*P* value	Adjusted *P* value
Age	≥60	54	1.489	1.152	2.054	0.043*	0.258
	<60	73	1.113	0.809			
Gender	Female	57	1.25	1.05	–0.226	0.821	1.000
	Male	70	1.291	0.933			
Blast cell	High	65	1.312	0.902	0.453	0.652	1.000
	Low	62	1.232	1.068			
Bone marrow blast cell	High	64	1.351	1.094	0.907	0.366	1.000
	Low	63	1.193	0.858			
Hemoglobin	High	102	1.229	0.882	–0.794	0.434	1.000
	Low	25	1.451	1.326			
Leukocyte	High	64	1.47	1.16	2.326	0.022*	0.154
	Low	63	1.072	0.718			
FAB	M3	13	0.457	0.341	–6.863	0.000***	0.000
	non-M3	114	1.366	0.99			
Cytogenetics risk	Favorable + Normal	100	1.266	1.015	–0.164	0.870	1.000
	Poor	27	1.298	0.874			

*p < 0.05, ***p < 0.001.

### Potentially Related Pathway Analysis for the Signature

**FIGURE 3:**
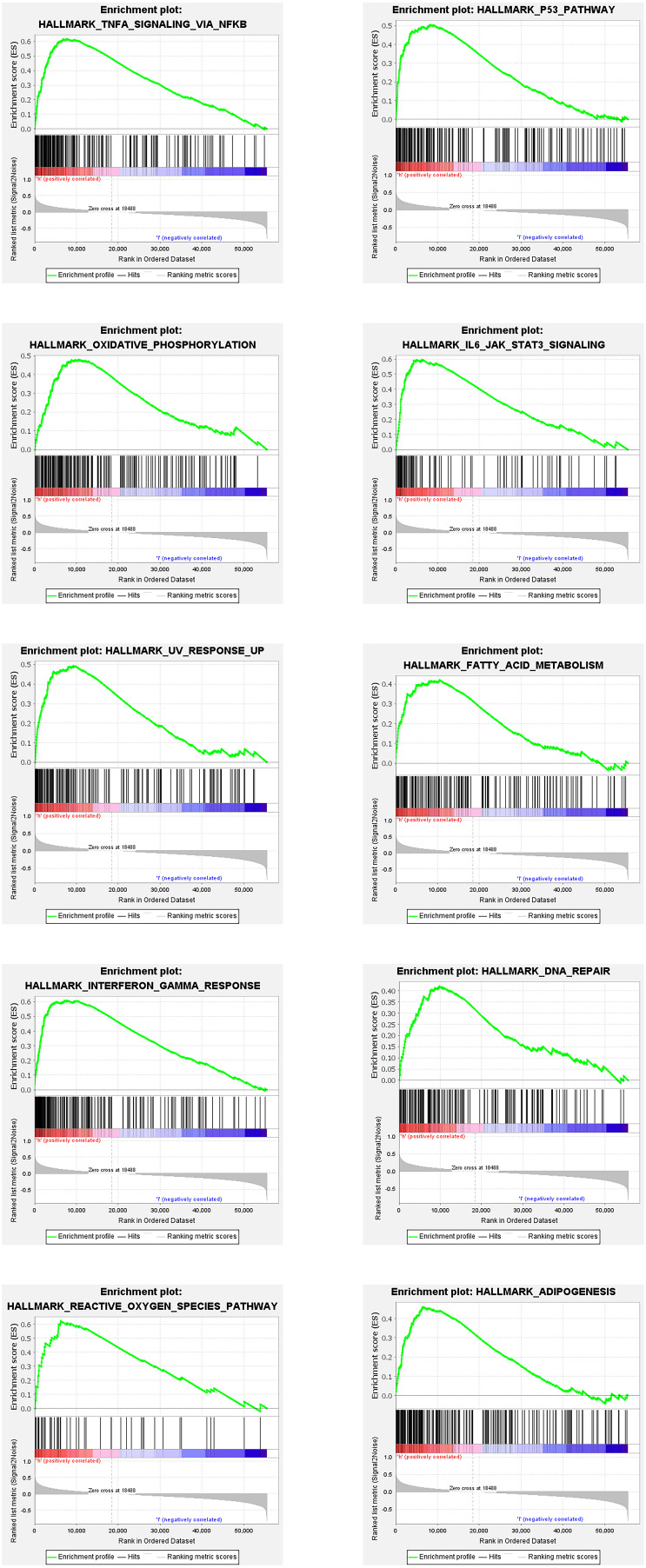
Top 10 enrichment plots from gene set enrichment analysis (GSEA).

The enrichment of differentially expressed genes in two groups (high risk and low risk) was assessed using GSEA to investigate the potentially related pathways of the signature. 20 related pathways based on *p* value < 0.05 and FDR < 0.25 are listed in Table 4. The top 10 pathways are shown in Figure 3.

**TABLE 4:** The results of Gene set enrichment analysis.

Pathway	NES	*P* value	FDR
HALLMARK_REACTIVE_OXYGEN_SPECIES_PATHWAY	2.27	0.000	0.012
HALLMARK_ADIPOGENESIS	1.99	0.006	0.054
HALLMARK_UV_RESPONSE_UP	1.93	0.004	0.066
HALLMARK_OXIDATIVE_PHOSPHORYLATION	1.91	0.041	0.054
HALLMARK_P53_PATHWAY	1.90	0.002	0.047
HALLMARK_FATTY_ACID_METABOLISM	1.86	0.008	0.0.53
HALLMARK_IL6_JAK_STAT3_SIGNALING	1.86	0.014	0.048
HALLMARK_TNFA_SIGNALING_VIA_NFKB	1.85	0.006	0.043
HALLMARK_INTERFERON_GAMMA_RESPONSE	1.84	0.027	0.043
HALLMARK_DNA_REPAIR	1.83	0.032	0.039
HALLMARK_COMPLEMENT	1.83	0.012	0.036
HALLMARK_CHOLESTEROL_HOMEOSTASIS	1.80	0.008	0.039
HALLMARK_PEROXISOME	1.76	0.010	0.049
HALLMARK_COAGULATION	1.75	0.012	0.046
HALLMARK_APOPTOSIS	1.70	0.017	0.06
HALLMARK_ALLOGRAFT_REJECTION	1.70	0.041	0.057
HALLMARK_INFLAMMATORY_RESPONSE	1.67	0.031	0.057
HALLMARK_IL2_STAT5_SIGNALING	1.65	0.031	0.064
HALLMARK_XENOBIOTIC_METABOLISM	1.62	0.025	0.072
HALLMARK_MYOGENESIS	1.56	0.026	0.090

## Discussion

Several autophagy related lncRNA signatures predicting the survival of patients have been proven in several cancers, including ovarian cancer, colorectal cancer, hepatocellular carcinoma, bladder urothelial carcinoma, breast cancer, colon adenocarcinoma, and glioma (Luan et al., 2019; Meng et al., 2020; Sun et al., 2020; Wei et al., 2020; Wu et al., 2020; Zhou et al., 2020; Li et al., 2021). However, autophagy related lncRNA prognostic signature has not been investigated in AML. This study aimed to establish an autophagy related lncRNA signature that might affect the survival of AML patients.

At first, a total of 866 lncRNAs were identified as autophagy related lncRNAs. 21 lncRNAs were confirmed to affect the survival time of AML patients. Four of them were the best candidates for the construction of a prognostic signature. Among the four lncRNAs, three of them (MIR133A1HG, AL359715.1, and AL356752.1) have a good prognosis of survival, while the other lncRNA (MIRLET7BHG) shows a poor prognosis of survival. To date, these four lncRNAs have not been investigated in AML. Further studies are needed to confirm the prognostic function for AML patients. The construction of a prognostic signature is based on these four autophagy related lncRNAs. Patients were divided into a low and a high risk group according to the risk score in signature. The results showed that the high risk group had a worse probability of survival than the low risk group. Furthermore, the Cox regression and the ROC curve confirmed that this signature potentially is an independent prognostic factor for AML patients.

The GSEA results shows that 20 pathways might be related to the differences between the risk groups based on signature in AML. Some pathways such as P53, IL6/JAK/STAT3, TNFα, and IL2/STAT5 have been proven to be related to AML or other tumors (Rucker et al., 2012; Binder et al., 2018; Ni et al., 2020; Wang et al., 2020). Thus, this signature might affect the survival of AML patients by regulating these pathways.

## Conclusion

The signature containing four autophagy related lncRNAs is an independent prognostic factor for AML patient’s survival time. These lncRNAs and their signature might be novel biomarkers for prognosis. Some tumor related pathways might be the potential mechanisms of the signature for the survival of AML patients. However, further investigations in AML are necessary to know the performance and the exact mechanism of lncRNAs in the signature in a clinical setting.

## Data Availability Statement

Publicly available dataset was analyzed in this study. The data was downloaded from in TCGA database.

## Author Contributions

YW, CZ, and ZW designed the article. YW and CZ collected and evaluated the data and wrote the first draft of the manuscript. ZW, SZ, XY, JL, FT, and JZ reviewed the manuscript. All authors contributed to the interpretation of the results, wrote the final draft of the manuscript, and read and approved the final version of the manuscript.

## Conflict of Interest

The authors declare that the research was conducted in the absence of any commercial or financial relationships that could be construed as a potential conflict of interest.

## Footnotes

1 https://xenabrowser.net/datapages/

2 http://autophagy.lu/clustering/index.html

## Abbreviations

SD: Standard deviation
FDR: false discovery rate
GSEA: gene set enrichment analysis
AML: acute myeloid leukemia
HR: hazard ratio
KEGG: Kyoto encyclopedia of genes and genomes
NES: normalized enrichment score
OS: overall survival
TCGA: the cancer genome atlas
lncRNA: long non-coding RNA.
